# Management of Sepsis

**Published:** 1960-07

**Authors:** Herbert K. Bourns

**Affiliations:** Surgeon-in-charge of the Casualty Department, Bristol Royal Infirmary


### MANAGEMENT OF SEPSIS

BY

HERBERT K. BOURNS, F.R.C.S.

*Surgeon-in-charge of the Casualty Department, Bristol Royal Infirmary*

Infection requires a host, an organism, and some other factor. This conditioning factor is unrecognised in many cases, but if sought in each case it often comes to light. It is important to look for it, because if we know what has started the process of sepsis, we may be able to remove it.

### THE ORGANISM

Many patients are carriers of a virulent organism, particularly the staphylococcus. The common site for this is in the nose. Thus in a recent investigation in Bristol it was found that of people who had not been in contact with a hospital for at least a year, over 40 per cent were nasal carriers of staphylococci[1]. Other common sites for harbouring pathogenic organisms are the perineum and throat. Most nasal carriers are also skin carriers, so it is easy to see how these patients can be infected by their own organisms. Recurrent boils or abscess formation should always lead one to swab these areas. The removal of the reservoir of the germs will usually stop further abscess formation.

In Birmingham in 1956, 95 per cent of septic fingers were due to staphylococci[2]. In the Bristol Royal Infirmary in 1957, 90 per cent were due to this organism and 10 per cent due to streptococci[1]. (These figures refer to infected fingers from which pus was available for culture.) The results in Bristol further supported a clinical impression that the *phage* type of the staphylococcus was related to the severity of the infection. Thus the troublesome Phage Type 80 *Staphylococcus* which was first isolated in 1953 by Rountree and Freeman (1955) in Australia[3] has enhanced virulence, and so infection with it leads to sloughs and tissue damage with considerable toxaemia.

A little boy was admitted with a partial-thickness burn of about twenty per cent. His initial course was satisfactory. There was a sudden deterioration in his condition and he became unconscious. Meningeal infection was suspected but lumbar puncture did not confirm this. The burnt surface was infected and the cause of the severe general reaction was shown when Type 80 Staphylococci were grown on culture. He rapidly improved when erythromycin was given in large doses.

The "phage types" which are resistant to more than one antibiotic are usually more virulent. Thus the spread of resistant organisms may lead to increase in the incidence of more serious infections.

### LOW RESISTANCE

Intercurrent diseases may lower the resistance either of a patient as a whole, or of his tissues locally. General conditions, such as diabetes mellitus, have to be looked for in all cases with infection and particularly in patients who appear to be healthy.

Healthy patients are more susceptible after an operation. "The increased congregation of more susceptible hosts" is probably one of the reasons for the increase in staphylococcal infections in hospital[4]. Since patients nowadays are discharged from hospital so early in their recovery period, this susceptible state persists into home life, and in this way some patients develop infections from their own home organisms which normally would not affect them.

### LOCAL FACTORS

Local trauma, with haematoma formation and divitalized tissue even if only present for a short time, may lead to the formation of an abscess. The conservative treatment of haematomata always carries the risk of infection, because organisms gain access to this extravasated blood. Moreover, fat necrosis as the result of contusion is much more common than is generally recognized; it gives rise either to immediate fluctuation and abscess formation, or to organization, fibrosis, and the appearance of an unexplained "tumour". This "tumour" is a firm and painless lump which may appear many months after the original trauma.

Neuropathy and vascular diseases have also to be considered.

Recently a patient had a "septic finger" for two years, despite all efforts to produce healing, because she had a lesion of the spinal cord affecting her sensation.

Peripheral vascular disease of all types should be considered, especially when there is infection of the hands or feet. If the circulation is inadequate chronicity of the infection is usual; but the real danger is the onset of gangrene of the tips of either fingers or toes. Whole toes or parts of fingers may have to be amputated in order to limit the infection to a region where the body defences are more adequate.

### BONE DISEASE

Bone disease is a potent source of trouble in infection. A sequestrum may form when the blood supply to the bone is inadequate, and as a result the process will not heal until the dead bone has separated and has been extruded. This is a troublesome feature in pulp space infections of the fingers; but it is less common now that incisions are correctly placed.

### BREAST ABSCESS

In breasts, the site of an abscess is very often a lobule with retained milk. Either weaning or inefficient feeding may be the cause, and either lack of hygiene or cracked nipple, or both, the precipitating factor. The complete emptying of all segments of the breast, at least once a day, will reduce the incidence of breast abscesses. More vigorous measures may help to resolve an abscess that has begun to form.

A mother was sent to hospital with a large indurated area in the lower half of the left breast. She had had five daily injections of a mega-unit of crystalline penicillin. She was weaning her baby and had been advised to use firm support for her breasts and only to empty them once a day. The treatment advised by the hospital was to stop all antibiotics, cease compression and to empty her breast three or four times a day. Within two weeks, the "abscess" had disappeared and the breasts became dry at about the same time. Much of this lump may well have been due to a galactocele and the use of a breast pump enabled the congestion to be removed, so that the affected area drained properly. The patient was saved pain and discomfort by this simple treatment.

### MIXED INFECTIONS

Chronic infections may become secondarily infected. This secondary infection will not clear up until the primary one is dealt with. Two recent cases illustrate this point:

A young boy had a large patch of infection on his forehead for many months. In hospital ringworm was demonstrated and when this had been treated the pus quickly cleared up.

Tuberculous lymphadenitis is often a cause of persistent sepsis, even when a pyogenic organisms has been identified.

Thus, a little girl had an abscess in her left groin; this was incised and she was discharged to the care of her own doctor. After some months, however, she was sent back with a granulating wound and profuse purulent discharge, for opinion as to what further antibiotic might be used. Excision of the wound and a diseased gland at the base of the wound revealed the cause of the trouble, for tubercle bacilli were demonstrated. The condition quickly cleared with excision of the affected gland, together with appropriate chemotherapy.

### EFFECT OF DRESSINGS

The type of dressing used may cause an infection either to persist or to spread. Gauze impregnated with soft paraffin is greasy, and does not facilitate the escape of wound exudate, nor does it allow the skin around the wound to be cleansed without the use of a fat solvent. The ulcer and the surrounding skin become macerated and so healing is delayed. Watson[2] found that changing from dry dressing to vaseline gauze delayed healing time by an average of two days in septic fingers. "Carbonet" has superseded both dry dressing and vaseline gauze and I have found it to be one of the best of the nonadherent sterile dressings[5]. It is water-soluble and so allows the wound to "breathe".

Cod liver oil impregnation of a wound leads to delay in healing because the tissues take up the large droplets and cannot dispose of them. The absorption leads to foreign body reaction. The wound edges become changed from healing granulation tissue to oil granulomata; this leads to poor healing and surrounding fibrosis.

A patient who was sent to the Bristol Royal Infirmary had sustained a deep wound above the right knee four months previously. The wound was unhealthy, the edges were fibrotic, and there was a persistent sero-purulent discharge. The wound was excised and the tissue removed was sent for histological examination. Many oil droplets were seen. The patient volunteered the information that for the first month after her injury the wound was packed with dressings impregnated with cod liver oil. The wound was sutured after excision, but it was another four months before it healed soundly. Our excision had not been wide enough, but this could not be helped, because the wound was so close to the knee joint.

### "ANTIBIOMA"

Antibiotic granulomas or "antibiomas" have been reported from time to time[6,7]. In such cases, the acute inflamation subsides but an indurated area remains for a long time. This is caused by the doctor's failure to diagnose the presence of pus, and his consequent failure to drain it; all abscesses should be opened and drained, even when the local and general reaction to antibiotic therapy appears to have been very satisfactory. Pus must always be drained, for if an abscess is not opened it may become inspissated and then slowly organises. This does not happen in all cases; the usual course of events when antibiotics are given without incision is for the abscess to persist as a fluctuant area; in time, the signs of acute inflammation return, unless the abscess bursts and discharges itself. This re-activiation may be caused by a fresh organism, or by the original one which multiplies afresh once the antibiotic is withdrawn. Antibiotics, of course, have a place in the treatment of sepsis, but they must not be employed as a substitute for drainage. They are of great value in combating systemic spread, but early incision must still be the order of the day if chronicity is to be avoided.

### TREATMENT

Treatment may be considered under two headings; treatment of the Patient and treatment of the Part.

*Treatment of the Patient*

To help a patient to combat any but the most trivial of infections, rest is essential. Many patients will not readily agree to rest when it is first suggested to them, but not infrequently they have to concede the necessity for this before the treatment has gone very far.

Pain should be relieved. But patients will frequently wait for medical advice before they will take such simple remedies as Aspirin or Tabs. Codeine Co., both of which are usually adequate in the average infection.

One should remind the patients to take more fluid by mouth. Many patients lose their appetite and so they do not have their normal meals. Since they do not wish to eat, they often omit to drink.

The patient must be given an explanation of his treatment and a guarded prognosis as soon as possible.

*Treatment of the Part*

Like that of the patient as a whole, this must commence with rest; a simple measure that is often overlooked. A sling is a necessity in most hand infections; rest in bed or in a chair with the foot elevated may be necessary for the lower limb.

Relief of pain is served by providing adequate dressings to support and protect the inflamed area. The frequent application of heat is comforting, whether by hot fomentation or heat lamp. Poulticing, on the other hand, has a number of serious drawbacks; the poultice tends to stick, it obscures the lesion from clinical inspection, and it causes scalding and maceration of the skin and superficial tissues. Patients, in order to prolong the effect, usually apply poultices far too hot. Unless changed frequently, poultices become "cold compresses".

If we wish to "draw" an abscess, glycerine and magnesium sulphate paste is one of the better local applications. Daily dressing is essential but there is little to be gained by increasing the frequency to more than twice in the twenty-four hours.

*Incision*

Relief of pain is achieved by relief of tension in the infected part. When swelling persists and becomes localized, suppuration is usually present. Incisions must be carried out as soon as fluctuation is present or when deep pus is suspected. It should be remembered that an abscess cavity is not a linear structure, so that, on incision, the cavity must be explored either with a gloved finger or by sinus forceps. Once its limits have been defined, it must be "de-roofed", so that pus will not "pocket"; in this way the wound will not close until the abscess cavity has filled in. Therefore, incision is not just a stab under inadequate anaesthesia, but a planned operation in an anaesthetized patient, without any hurry, so that the surgeon can carry out a thorough inspection of the abscess cavity. Only in this way will adequate drainage be achieved and speedy healing attained.

I do not advocate "incision and suture" of an abscess. This form of treatment has been recommended mainly by Ellis of Leeds[8]. After incision the cavity is curretted and then when all pus and debris has been removed, the wound is stitched. Pressure is applied to prevent haematoma formation and antibiotics are given. This is stated to produce early healing and less scarring. This is a practice which has few advantages and many disadvantages. Firstly, I am sure that to be successful this treatment must be carried out only by an expert; in other words it cannot be applied generally. Secondly, it is based on the principle that the organism (which can only be obtained at the time of the incision) will be sensitive to whatever antibiotic is being used. However, we do not *know* the organism or its sensitivity until forty-eight hours have elapsed. Thirdly, the currettage of the abscess cavity leads to disruption of the barrier already laid down round the abscess cavity, and to damage of defensive cells growing round the abscess. Fourthly, a stitched abscess does not heal any more quickly than an open abscess that is thoroughly drained, and so there is little difference in scarring.

If the infecting organism is sensitive to penicillin this antibiotic should be given. It is best given by intramuscular injection, and in the average case adequate dosage for a few days is better than low dosage for a long time. However, prolonged use of antibiotics is essential in bone, tendon, and joint infection. These tissues are all "specialized" and must be preserved at all costs if normal function is to be restored. Antibiotics may also be necessary in patients who are ill from other causes, such as diabetes, tuberculosis, nutritional disorders; or when they have had drugs such as corticoids. In recurring infections and in reparative processes, antibiotics may have to be given for a long period.

In a carbuncle, skin loss may be considerable. When the slough has been removed and a healthy granulating surface has been produced, the use of a skin graft should not be rejected for fear of infection. Identification of the type and sensitivity of the causative organism should be obtained, and then the defect should be covered with skin, while the appropriate antibiotic is given to cover the procedure.

*Restoration of function*

Healing by fibrosis as the result of granulation tissue will lead to loss of function and stiffness. Early restoration of movement is desirable. Movement, however, must not be encouraged until the infection is under control. One must persist with rest until the inflammatory process has settled completely. As soon as the infection has been localized and drained, the part will move easily. If movement is allowed too early, further spread of the infection will be promoted and chronicity will be produced. Apart from this, the patient's confidence may be undermined because early function will be painful.

In a recent case, a doctor had a boil on the right wrist. It was not very painful so he played a round of golf. Within twelve hours the surrounding oedema was testimony to the increase in his pain; there was lymphangitis of the arm and lymphadenitis of the axillary glands together with a raised temperature demonstrating the general toxaemia. Twenty-four hours rest, without antibiotics, caused a return to the previous localized state.

In infections of the hand immobilisation in the position of physiological rest does not have any permanent unpleasant results if early healing is attained. Even tendon sheath infections and exposure of the tendons themselves may be overcome if the wound is closed early. Scar tissue dissolves in time, so that one must not be discouraged by early lack of mobility. The final result will be much better if rest is maintained until healing is complete.

*Dressings*

The dressing must be sterile. As mentioned above, it should be non-greasy and non-irritant. "Carbonet" is one of the best at present, because it is easily sterilized and harmless to the tissues. Dressings should only be changed when the volume of discharge makes this necessary. Dressings are intended as a protection for the wound; they should therefore be large enough for this purpose. Constriction should always be avoided; this particularly applies to the use of "Tubegauz" with which the unskilled may easily produce a constriction at the edge of the dressing by excessive twisting or pulling during the application of the bandage. (The manufacturers of this bandage are well aware of this danger and warn against it in all their literature.)

Dressings must be kept out of water. If by accident they become wet, then they should be changed. On the other hand, enclosure in a water-tight cover is bad because, in the first place, many covers are so inefficient that moisture will inevitably get in, and secondly the cover prevents the natural heat of the part from promoting the drying of the moist dressings. Incidentally, many do not realize that a watery dressing only remains wet for about half an hour unless covered by a water-proof cover. Yet if dressings are covered in this way, maceration is soon produced so that healing is delayed.

In conclusion, management of septic lesions should consist of rest for the patient and the part, antibiotics, adequate incision and drainage, search for any possible cause, promotion of early healing, and finally mobilisation.

REFERENCES

[1] James, W. and Adler, V. G. To be published.
[2] Williams, J. A., Meynell, M. J. and Watson, A. B. (1956). *Brit. med. J. i*, 716.
[3] Rountree, P. M. and Freeman, B. M. (1955). *Med. J. Austr.* **2**, 157.
[4] McDermott, W. (1956). *Brit. med. J. ii*, 837.
[5] Bourns, H. K. (1958). *Lancet ii*, 420.
[6] Stammers, F. A. R. (1953). *Brit. med. J. i*, 272.
[7] Mills, C. P. (1953). *Brit. med. J. i*, 1427.
[8] Ellis, M. (1951). *Lancet i*, 1038.

